# Tumor Mutational Burden in Breast Cancer: Current Evidence, Challenges, and Opportunities

**DOI:** 10.3390/cancers15153997

**Published:** 2023-08-07

**Authors:** Romualdo Barroso-Sousa, Jana Priscila Pacífico, Sarah Sammons, Sara M. Tolaney

**Affiliations:** 1 Dasa Institute for Education and Research (IEPD), Brasilia 71635-580, DF, Brazil; 2 Dasa Oncology, Hospital Brasilia, Brasilia 71635-580, DF, Brazil; 3 Department of Medical Oncology, Dana-Farber Cancer Institute, Boston, MA 02215, USA; 4 Breast Oncology Program, Dana-Farber Brigham Cancer Center, Boston, MA 02215, USA; 5 Harvard Medical School, Boston, MA 02115, USA

**Keywords:** tumor mutational burden, breast cancer, immune checkpoint inhibitors

## Abstract

**Simple Summary:**

The tumor mutational burden (TMB) can be defined as the number of somatic mutations per megabase of the sequenced genome. It correlates with tumor neoantigen burden, T cell infiltration, and response to immune checkpoint inhibitors in many solid tumor types. In addition, TMB has been used as a biomarker of benefit to pembrolizumab in a tissue-agnostic manner; however, in breast cancer limited data exist regarding the true role of this biomarker in clinical practice. This review describes current knowledge about TMB in breast cancer and its potential role as a predictive biomarker of immunotherapy in the advanced setting. We also discuss the need for additional work to better understand how to integrate TMB in clinical practice, including the establishment of the best genomic tool to determine TMB, the ideal TMB cutoff, and the optimal immunotherapy regimen.

**Abstract:**

Tumor mutational burden (TMB) correlates with tumor neoantigen burden, T cell infiltration, and response to immune checkpoint inhibitors in many solid tumor types. Based on data from the phase II KEYNOTE-158 study, the anti-PD-1 antibody pembrolizumab was granted approval for treating patients with advanced solid tumors and TMB ≥ 10 mutations per megabase. However, this trial did not include any patients with metastatic breast cancer; thus, several questions remain unanswered about the true role of TMB as a predictive biomarker of benefit to immune checkpoint inhibitor therapy in breast cancer. In this review, we will discuss the challenges and opportunities in establishing TMB as a predictive biomarker of benefit to immunotherapy in metastatic breast cancer.

## 1. Introduction

Breast cancer is the second-leading cause of cancer death in women in the United States [1] and the leading cause worldwide [2], with most deaths resulting from metastatic breast cancer (MBC). Approximately 30% of women who are initially diagnosed with stage I-III disease will develop a distant recurrence, and 6–10% of breast cancer patients will be diagnosed with de novo metastatic disease [3]. Despite the emergence of new therapies that extend survival, MBC is still considered incurable [4], and novel treatments are needed.

The use of immune checkpoint inhibitors (ICI) in combination with chemotherapy has improved progression-free survival (PFS) and overall survival (OS) in a subset of patients with metastatic triple-negative breast cancer (mTNBC) [5,6,7]. However, these benefits are mainly restricted to treatment-naïve patients with programmed death-ligand 1 (PD-L1)-positive disease. Furthermore, patients with hormone receptor-positive (HR+) MBC have not yet derived benefit from immunotherapy [8,9,10]. These findings highlight the need to identify the molecular mechanisms that mediate the immunosuppressive tumor microenvironment in HR+ MBC and PD-L1-negative mTNBC. Additional biomarkers beyond PD-L1 status are needed to identify patients who might derive the most benefit from ICIs.

Tumor mutational burden (TMB) can be defined as the number of somatic mutations per megabase of the sequenced genome. It correlates with tumor neoantigen burden, T cell infiltration, and response to ICIs in many solid tumor types [11,12,13,14,15,16,17]. In the multicenter, open-label, phase II KEYNOTE-158 study of patients with advanced solid tumors, the anti-PD-1 antibody pembrolizumab produced an objective response rate (ORR) of 29% (95% confidence interval [CI]: 21–39%) in patients with TMB ≥ 10 mutations per megabase of DNA (mut/Mb) compared to 6% (95% CI: 5–8%) in patients with TMB < 10 mut/Mb [18]. While data from the KEYNOTE-158 study led to the approval of pembrolizumab to treat patients with TMB-high status (≥10 mut/Mb as determined using the FoundationOne CDx assay) regardless of tissue of tumor origin (tumor tissue-agnostic approval), this trial did not include any patients with MBC. Thus, several questions remain unanswered about the true role of TMB as a predictive biomarker of benefit to ICI therapy in breast cancer. In this review, we will discuss the challenges and opportunities in establishing TMB as a predictive biomarker of benefit to immunotherapy in MBC.

## 2. Prevalence of High TMB in Breast Cancer 

Compared to other malignancies, breast cancer has an intermediate TMB [12]. Previously, our group analyzed data from 3966 breast tumors included in eight cohorts. The study reported a median TMB of 2.63 mut/Mb [19]. Approximately 5% of all breast cancers had a TMB ≥ 10 mut/Mb, and metastatic tumors had a greater prevalence of high TMB than primary tumors (8.4% versus 2.9%). We also showed that hypermutated tumors had higher expression of CD8-positive T-cell effectors in the *GZMA* and *PRF1* genes leading to a greater immune cytolytic activity, and ultimately suggesting that these patients are more likely to respond to ICI therapies.

In terms of histology, it has been consistently shown that the prevalence of hypermutated tumors is significantly higher among metastatic invasive lobular carcinomas compared to metastatic invasive ductal carcinomas (17.0% versus 7.8%) [19,20], which raises the question of whether a subgroup of those tumors could be treated with immunotherapy-based regimens. Recently, the GELATO trial investigated the role of weekly carboplatin as immune induction plus atezolizumab exclusively in patients with metastatic invasive lobular carcinoma [21]. Although the trial met its primary endpoint, with four partial responses out of 23 patients, only one out of 23 patients had a TMB ≥ 9 mut/Mb; therefore, further studies are needed to address this question specifically in patient populations with high TMB.

With regards to breast cancer subtypes, while triple-negative breast cancer (TNBC) has a higher median TMB than HR+ or HER2-positive breast cancers, the frequency of hypermutated tumors (≥10 mut/Mb) is similar among different subtypes [19,22,23].

## 3. Mutational Determinants of Hypermutation in Breast Cancer

Mutational processes in tumors can generate unique patterns of DNA alterations called mutational signatures. By analyzing such patterns, it is possible to identify the underlying specific mutagenesis process, including defective DNA repair, enzymatic modification of DNA, ultraviolet light damage, or tobacco smoke.

Given the fact that mismatch repair deficiency (MMRd) is associated with TMB-high status and is a validated predictive biomarker to select patients for using ICI, it was important to investigate whether it would be the major mutational process related to hypermutation in breast cancer, especially because MMR status can be assessed by the evaluation of MMR protein expression using immunohistochemistry. 

Additionally, because specific patterns of DNA alterations will generate distinct tumor neoantigens that will ultimately determine immunogenicity of the peptides presented to the immune system and the capacity of triggering an immune response following different immunotherapies, it is of interest to understand the mutational process related to hypermutation. In other words, the correlation between TMB and immunogenicity is dependent on the underlying mutational process [24,25,26,27]. For instance, while the UV signature and the APOBEC signature are strongly correlated with immune activation, the aging process signature is not.

In breast cancer, we and other groups have demonstrated that APOBEC dysregulation is the major mutational process associated with high TMB, found in approximately 60% of all hypermutated breast tumors [19,20,28]. APOBEC or apolipoprotein B mRNA-editing enzyme catalytic polypeptides are a family of cytidine deaminases that primarily deaminate viral genomes, leading to damage of these pathogens as part of the innate immune response [29]. However, besides protecting cells from viral infection, APOBEC enzymes can also induce significant mutagenic effects on host genomes causing primarily cytosine-to-thymine mutations. Besides breast cancer, APOBEC dysregulation has been shown to be a major driver of mutations in bladder cancer [29,30,31]. Other dominant signatures found in hypermutated breast tumors are MMRd (around 36%) followed by homologous recombination deficiency associated with *BRCA1/2* mutations (1%) and the one associated with dysregulation of DNA polymerase epsilon (*POLE*) presented in 4% of these tumors [19,28]. One practical consequence of this finding is that searching only for MMRd with immunohistochemistry is not adequate to identify the majority of patients with TMB-high breast cancer.

Notably, several groups have shown that prolonged responses can be achieved with the use of ICI in patients with TMB-high breast cancer associated with APOBEC dysregulation [19,28,32].

## 4. Prognostic Role of TMB in Breast Cancer 

Previously, Samstein et al. [33] have shown that TMB was not associated with OS in a cohort of 860 patients with any subtype of breast cancer treated with non-ICI therapies. Using the same dataset, we found a similar finding examining only patients with TNBC from the same institution. In agreement, a recent meta-analysis using data from 16 cohorts with 1722 patients concluded that TMB is not prognostic in breast cancer in general, and that high TMB may be associated with longer survival only in patients treated with ICI-based regimens [34].

Moreover, emerging data suggest that high baseline TMB is significantly associated with no clinical benefit and shorter PFS among patients with metastatic HR+/HER2-negative breast cancer treated with first-line endocrine therapy plus CDK4/6 inhibitors [35]. Notably, these hypermutated tumors are enriched for alterations in signaling pathways previously implicated in driving endocrine therapy and CDK4/6 inhibitor (CDK4/6i) resistance, including PI3K-AKT and RTK-RAS. Another study showed that a dominant APOBEC signature that is enriched in hypermutated tumors is associated with inferior outcomes among patients treated with endocrine therapy plus CDK4/6i [32]. Altogether, these data suggest that patients with primary resistance to CDK4/6i could be candidates for clinical trials evaluating the benefit of ICI-based therapies, even those with PD-L1-negative tumors.

## 5. TMB and the Benefit of Chemoimmunotherapy in MBC

Previously, our group analyzed a cohort of patients treated at Dana-Farber Cancer Institute with different regimens containing anti-PD-1/PD-L1 agents given as monotherapy, with chemotherapy, or with targeted agents. We observed a positive association of high TMB with longer PFS and OS among patients with mTNBC following treatment with ICI, independent of clinical factors and PD-L1 status [36]. As mentioned above, the association between PFS and TMB was not observed among patients treated with chemotherapy only.

In the IMpassion130 study [5], atezolizumab combined with nab-paclitaxel showed improved PFS and clinically meaningful OS benefit versus placebo combined with nab-paclitaxel for patients with mTNBC with PD-L1 ≥ 1% expression on tumor-infiltrating immune cells. An exploratory analysis evaluated the association of TMB and outcomes in this trial [37]. Median TMB was 4.39 mut/Mb (range, 0–46.51), and there was no association found between TMB and PD-L1 status, but higher TMB levels were associated with improved PFS and OS in the atezolizumab versus placebo arm only in patients with PD-L1-positive tumors. Data on the frequency of patients with TMB ≥ 10 mut/Mb or the benefit specifically in this population was not reported [37]. To date, we do not have information about TMB and responses in patients included in the KN355 study, the phase III trial that established the use of pembrolizumab combined with chemotherapy for patients with PD-L1-positive mTNBC [38].

In the setting of HR+/HER2-negative disease, our group led a randomized phase II study comparing the efficacy of eribulin with or without pembrolizumab. Patients had received endocrine therapy and between 0–2 prior lines of chemotherapy regimens for metastatic disease [39]. After a median follow-up of 25.8 months, the trial was declared negative, and neither median PFS nor OS differed in the treatment arm. Out of 88 patients included in this study, whole-exome sequencing data from 50 patients were available for analysis. Overall, the median nonsynonymous TMB was 2.5 mut/Mb, and six (12%) patients had a TMB ≥ 10 mut/Mb. The dominant mutational signature in most tumors was related to aging (98% related to aging), while all six tumors with TMB ≥ 10 mut/Mb had mutational signatures related to APOBEC. In this study, we did not find any association between TMB or APOBEC signature and benefit (objective response or survival outcomes) in the overall population or in the ICI-treated arm. Notably, only 4 out of the 27 patients treated in the ICI-treated group had high TMB precluding a powered analysis regarding the predictive role of high TMB and ICI efficacy in our study. 

## 6. TMB and the Benefit of ICI without Chemotherapy in MBC

The KEYNOTE-119 trial [40] compared pembrolizumab monotherapy to chemotherapy in 622 patients with pretreated mTNBC. TMB data were available for 253 out of 601 (42.1%) treated patients (pembrolizumab, n = 132; chemotherapy, n = 121). Although the results of an exploratory analysis about TMB and survival must be interpreted with caution due to the small sample size (only 26 out of 253 patients or 10.3% had high TMB (TMB ≥ 10 mut/Mb)), data from this study suggest that patients identified as TMB-high have a greater survival benefit with pembrolizumab compared with chemotherapy (OS hazard ratio 0.58 [0.21–1.57]). 

In the phase II KEYNOTE-086 trial, which evaluated the use of pembrolizumab monotherapy in patients with advanced TNBC both in first line (only for patients with PD-L1 positive defined as CPS score ≥ 1) and in second or later lines (for patients previously treated with chemotherapy independent of CPS score), data showed a significant association between higher TMB (as a continuous variable) and increased ORR, PFS, and OS compared to the lower TMB group [41]. Notably, there was no correlation between TMB and the other immune biomarkers evaluated (PD-L1, stromal tumor-infiltrating lymphocytes, CD8 density, and immune signature). Similarly, a pan-cancer analysis that included TNBC patients also suggested an association between TMB and higher ORR. The study also showed a poor correlation between TMB and PD-L1 and TMB and the same immune gene signature. Additionally, as reported in the NIMBUS study [42], only 14% of patients included in the trial had PD-L1-positive tumors, and PD-L1 positivity was not associated with improved ORR. Altogether these data suggest that TMB and PD-L1 are markers of different and potentially complementary steps of the cancer-immune cycle and could be used as complementary biomarkers.

In the metastatic setting, the phase II basket TAPUR trial [43] included a small number of patients (n = 28) with MBC and high TMB (TMB ≥ 9 mut/Mb) treated with pembrolizumab monotherapy. TMB-high status ranged from 9 to 37 mut/Mb, disease control was obtained in 37% of patients, the ORR was 21% (95% CI 8–41%), and the median PFS and OS were 10.6 and 30.6 weeks, respectively. No association was found between increasing TMB and longer PFS. Data from a retrospective analysis of 62 patients with mTNBC also suggest immune checkpoint blockade therapy alone may be sufficient to treat TMB-high tumors [36]. Among patients treated with anti-PD-1/L1 therapies, high TMB (18%) was associated with significantly longer PFS (12.5 versus 3.7 months; *p* = 0.04).

While these data suggest single-agent checkpoint inhibitor therapy has activity in metastatic solid tumors with high TMB, there are also data to suggest that dual checkpoint inhibition could potentially lead to even greater activity. Preclinical and clinical data support the idea that the anti-PD-1 nivolumab and the anti-CTLA-4 antibody ipilimumab act synergistically to promote T-cell antitumor activity through complementary mechanisms of action [44,45,46]. Furthermore, data from the CHECKMATE-142 study suggest that the combination of nivolumab and ipilimumab has better efficacy outcomes and is associated with a numerically higher response rate than nivolumab monotherapy in patients with DNA mismatch repair-deficient/microsatellite instability-high metastatic colorectal cancer [47,48]. More recently, nivolumab plus low-dose ipilimumab was approved as first-line treatment for patients with DNA mismatch repair-deficient/microsatellite instability-high metastatic colorectal cancer and metastatic non-small cell lung cancer expressing PD-L1 ≥ 1% using an approved FDA test [49,50].

Based on this rationale, we conducted the phase II, single-arm NIMBUS study [42], which included and treated 30 patients with HER2-negative MBC and high TMB (≥9 mut/Mb) with a combination of nivolumab (3 mg/Kg every 2 weeks) plus low-dose ipilimumab (1 mg/Kg every 6 weeks). The confirmed ORR was 16.7%, and the primary endpoint of this study was met. No new toxicities were identified for this combination, and no grade 4 or 5 adverse events were reported. Notably, the analysis of the key prespecified secondary endpoint showed that patients with TMB ≥ 14 mut/Mb achieved an ORR of 60% (three out of five patients), while those with TMB ≥ 9 and < 14 mut/Mb had an ORR of 8% (two out of twenty-five patients). In addition, patients with TMB ≥ 14 mut/Mb experienced significantly longer PFS and OS compared to patients with lower TMB.

Important differences between the study populations of TAPUR and NIMBUS are highlighted in Table 1. The proportion of patients with estrogen receptor-positive MBC was 70% in NIMBUS and 43% in TAPUR; only 14% of patients included in NIMBUS had PD-L1-positive tumors (a tumor presenting a CPS score ≥ 10 was classified as PD-L1 positive), while this information is unknown in TAPUR. Furthermore, it is notable that during the enrollment of the TAPUR study, there was no approval for ICI in MBC while NIMBUS accrued patients mostly after the approval of atezolizumab in MBC. Therefore, in NIMBUS there was an enrichment of patients who did not meet the requirements for using ICI in combination with chemotherapy (estrogen receptor-positive tumors and PD-L1-negative TNBC). Conversely, the TAPUR study included more heavily pretreated patients and more patients with an Eastern Cooperative Oncology Group performance status of 1. 

Responses during the NIMBUS study have been durable, with some patients remaining progression-free for more than two years after stopping treatment. Other studies using the combination of nivolumab and ipilimumab have also shown durable responses in MBC, particularly in patients with metaplastic MBC [51]. Superior activity of the combination of anti-CTLA-4 plus anti-PD-1 versus anti-PD-1 alone might be due to an increase in the ratio of cytotoxic/regulatory T cells within the tumor [52,53] and augmented release of IFN-γ and TNF-α. 

While the NIMBUS study supports the use of ICIs for patients with MBC and high TMB, it does not answer the question of whether dual checkpoint inhibition is better than pembrolizumab monotherapy. Another limitation is the fact that the determination of TMB for eligibility for these two studies allowed use of different genomic panels, and central testing was not required to meet eligibility.

## 7. TMB and Benefit of Immunotherapy in the Neoadjuvant Setting

Currently, the addition of pembrolizumab to neoadjuvant chemotherapy is the standard of treatment for patients with stage II-III TNBC. In a predefined analysis of the neoadjuvant trial GeparNuevo, Karn et al. [54] investigated the association of TMB and clinical outcomes in this study and showed that TMB may have clinical utility in predicting pathologic complete response (pCR) in early-stage TNBC as well. Among the 149 early-stage TNBC included in GeparNuevo who received neoadjuvant durvalumab and chemotherapy or chemotherapy alone, the median TMB reported was 1.52 mut/Mb and was significantly higher in patients with pCR (median 1.87 versus 1.39, *p* = 0.005). Notably, TMB demonstrated independent value for pCR prediction in multivariate analysis.

## 8. Conclusions

TMB has been used as a cancer tissue-agnostic predictive biomarker of benefit to pembrolizumab; however, there are only few data about its definitive role in breast cancer. Most cases of hypermutated breast cancers arise from APOBEC dysregulation. Although data generated by TAPUR and NIMBUS studies support the use of immunotherapy in breast cancer, more work is needed to establish the best TMB cutoff and the best immunotherapy regimen to be used in breast cancer.

## 9. Future Directions

Before we can fully develop TMB as a predictive biomarker of immunotherapy benefit in breast cancer, we must address some remaining challenges (Table 2) [55,56]. 

First, it is critical to recognize that there is a spectrum of actionability for any target in oncology, ranging from histology-specific targets to histology-agnostic targets. Therefore, the level of actionability is dynamic and dependent upon the clinical development of new agents and drug combinations [57]. 

With regards to TMB, as previously discussed, the dominant mutational process that causes hypermutation can impact the neoantigen immunogenicity and the response to ICI. Moreover, previous studies in other cancer subtypes have demonstrated that specific genomic alterations can also contribute to immune evasion and impede immunotherapy, including b2-microglobulin mutations that can cause impairment of antigen presentation by disrupting MHC class I [58,59]; *JAK1/2* [59] and *STK11* [60] mutations; WNT/b-catenin pathway alterations [61,62]; and *PTEN* loss [63]. On the other hand, PD-L1 (*CD274*) gene amplification and *BRCA2* mutations have been associated with higher responses to ICI [64,65]. 

Data on breast cancer are scarce, and a previous retrospective study from our group has associated *PTEN* loss with lack of response to immunotherapy in TNBC [36]. Recently, using a syngeneic genetically engineered mouse model of invasive breast cancer driven by ablation of both *PTEN* and *TRP53*, Bergholz et al. [66] demonstrated that in tumors with PTEN loss, the kinase PI3Kβ promotes immune evasion by upregulation of *STAT3*. Moreover, both genetic inactivation or pharmacological inhibition of PI3Kβ triggered a robust anti-tumor immunity response and synergized with immunotherapy to inhibit tumor growth. Exploratory analysis from the randomized phase II SAFIR02-BREAST IMMUNO study, which included patients with HER2-negative MBC whose disease did not progress after 6–8 cycles of chemotherapy and randomized them to either durvalumab or maintenance chemotherapy, identified PD-L1 amplification as a potential biomarker of benefit to durvalumab specifically in the TNBC population [67]. 

Given the prevalence of TMB-high status in patients with MBC (around 10%), it would be ideal to perform prospective and randomized studies to address whether TMB can be useful as a predictive biomarker of response or resistance to immunotherapy, specifically in this population. 

Second, there are important issues related to analytical aspects of the clinical validation of TMB as a biomarker, including the ideal cutoffs, the best genomic platform to evaluate TMB, and the disease setting and specific regimens used by the population of interest.

The absence of consensus on the definition of TMB cutoffs for patient selection is still a problem. In agreement with the results of the NIMBUS study that showed that patients with a TMB ≥ 14 mut/mb had a higher ORR (60%) than patients with TMB ≥ 9 and <14 mut/Mb (8%), results of the phase IIa multi-basket MyPathway study [68] demonstrated that atezolizumab monotherapy had promising, durable clinical activity (ORR 38.1%) across a variety of advanced solid tumor types in patients with TMB ≥ 16 mut/Mb, but had limited activity (ORR 2%) among tumors with TMB ≥ 10 and < 16 mut/Mb. Of the seven breast cancer patients in this cohort with TMB ≥ 16 mut/Mb, one patient with HR+, HER2-negative MBC had a response. Other studies suggest that ideal MBC TMB-high cutoff for response is likely higher than 10 mut/Mb [32]. While TMB was initially studied as a biomarker by performing whole-exome sequencing, next-generation sequencing targeted panels can estimate TMB in a time-effective and cost-effective way [17]. However, the lack of harmonization between the different assays and methodologies used to calculate TMB is an important barrier to implementing TMB in clinic. Differences in panel size, lists of genes covered by the panel, and bioinformatic pipelines bring variability in TMB estimates across different panels. Furthermore, it is critical to recognize that the current development of algorithms for variant calling and TMB calculation has been performed and validated in cohorts predominantly composed of White patients with European ancestry. Thus, the generalizability of the TMB cutoff used in non-White populations is questionable. Studying two independent clinical cohorts, Nassar et al. [69] found that tumor-only sequencing panels commonly overestimate TMB, especially in non-Europeans. While the erroneous TMB-high classification would be expected in 21% of individuals of European ancestry and in around 40% of Asian or African descendants. Efforts to harmonize TMB measurement are ongoing [70,71]. Moreover, there is a need to evaluate the concordance between tissue- and blood-derived genomic analyses and to explore the ideal cutoffs for classifying tumors as high TMB by using blood-derived assays and their correlation with tissue TMB [72,73,74,75].

Recently, data from patients who were screened for enrollment into CheckMate 848, (including 8.8% of samples from patients with breast cancer) a prospective phase II study evaluated the efficacy of nivolumab plus ipilimumab and nivolumab monotherapy in patients with advanced or metastatic solid tumors with high TMB, were used to evaluate the concordance between blood and tissue TMB [76]. Among the 1017 tissue and plasma sample pairs, the correlation between the two estimated TMB scores was identified (Spearman’s r, 0.48; *p* < 0.0001). Furthermore, 15.8% and 20.7% of samples had high tissue and blood TMB, respectively, (according the prespecified cutoff of 10 mut/Mb); the positive, negative, and overall percentage agreements between assays were 60%, 88%, and 84%, respectively. Notably, when only samples with plasma maximum allele frequency ≥ 1% were analyzed, TMB and positive percentage agreement were improved (TMB correlation (Spearman’s r, 0.54; *p* < 0.0001) and positive percentage agreement (66%)). Altogether, the data from this exploratory analysis suggest that data from paired biopsies are complementary and may be needed to correctly identify tumors with high TMB. Additionally, disease burden and plasma tumor fraction need to be considered to correctly interpret blood-derived assays. 

Finally, it is also important to mention that in other disease subtypes, such as non-small cell lung cancer, the association of high TMB and clinical benefit following ICI therapies remains controversial [77,78,79,80]. Of note is the heterogeneity of studies exploring TMB as a biomarker in clinical oncology, including differences in disease context (e.g., lines of therapy in the metastatic setting, metastatic versus early stage), and in the use of immunotherapy-based regimens, such as ICIs, given as monotherapy or the use of PD-1/PDL1 plus chemotherapy. It is important to remember that a biomarker is developed for a specific disease context and a specific therapeutic regimen.

Further development of TMB as a predictive biomarker can provide access to immunotherapy for patients with breast cancer subtypes other than PD-L1-positive TNBC. Given the numbers of patients with MBC and considering that around 10% of these patients have hypermutated tumors, this represents a large number of patients globally and highlights the need for further development. The use of TMB could select patients (including those with PD-L1-positive mTNBC) to be treated with a chemotherapy-free immunotherapy-based regimen, thereby sparing them from the toxicities of chemotherapy. The recent results of the phase II NICHE2 study [81] show an impressive pCR rate of 95% among patients with MMRd and colorectal cancer treated with a chemotherapy-free regimen containing nivolumab and highlights the potential to use immunotherapy without chemotherapy in patients with immunogenic tumors. Exceptional responders to chemotherapy-free immunotherapy-based regimens within breast cancer exist [42,43,82]. Further prospective clinical trials are needed to elucidate the optimal TMB cutoff and the best immunotherapy strategy in breast cancer.

## Figures and Tables

**Table 1:** Baseline demographics and clinical characteristics of patients included in the TAPUR and NIMBUS studies.

Characteristic	TAPUR (N = 28)	NIMBUS (N = 30)
**Age, median (range), years**	63 (36–78)	63.0 (36–72)
**Female, %**	100	100
**Race, White/Non-white, %**	75/25	90/10
**ECOG-PS 0/1, %**	36/64	73/27
**HR+/TNBC, %**	43/46	70/30
**Prior lines of CT in the advanced setting, median (range)**	3 (2–3)	1.5 (0–3)
0, n (%)	0	8 (27)
1, n (%)	0	7 (23)
2, n (%)	2 (7)	8 (27)
3, n (%)	26 (93)	7 (23)
**PD-L1 positive ^#^ (22C3 DAKO), %**	Not reported	14%
**TMB (mut/Mb), median (range)**	14 (9–37)	10.9 (9–110)
≥9–<14, n (%)	14 (50)	25 (83.3)
≥14–<20, n (%)	6 (22)	2 (6.7)
≥20, n (%)	6 (22)	3 (10)
Unknown	2 (6)	0 (0)
**Objective response rate, %**	21 *	16.7

* unconfirmed objective response; ^#^ patients with a tumor presenting a CPS score ≥ 10 were classified as PD-L1 positive. CT: chemotherapy; ECOG-PS: Eastern Cooperative Oncology Group performance status; HR+: hormone receptor-positive; mut/Mb: mutations per megabase; PD-L1: programmed death-ligand 1; TMB: tumor mutational burden; TNBC: triple-negative breast cancer.

**Table 2:** Factors influencing the development and clinical implementation of tumor mutational burden (TMB) as a useful biomarker in breast cancer.

Factors Influencing the Development and Clinical Implementation of TMB as a Biomarker	Explanation/Examples
Biological issues	
- Dominant mutational signatures	The mutational process leading to hypermutation influences the quality of neoantigens and, therefore, the immunogenicity of tumors. Thus, distinct mutational process differently influences the likelihood of benefit to immune checkpoint inhibitors.
- Specific genomic alteration	Specific tumor genomic alterations can impact both the tumor antigenicity, such as molecular alteration in genes related to the antigen-presentation machinery, as well as in genes that affect antitumor immunity, such as *PTEN* alterations.
Analytical issues	
- Use of different cutoffs for classifying a tumor as having high TMB	Lack of agreement about the ideal cutoff
- Use of different genomic platforms to evaluate TMB	Whole-exome sequencing versus large target-panels Blood versus tissue biopsies Germline-paired tests versus algorithm
- Population used for validation	Ethnic diversity Disease setting heterogeneity Use of different treatment regimens with or without chemotherapy
